# Epigenetics and genetics of hepatoblastoma: Linkage and treatment

**DOI:** 10.3389/fgene.2022.1070971

**Published:** 2022-11-30

**Authors:** Li-ran Zhu, Wanqun Zheng, Qun Gao, Tianping Chen, Zhu-bin Pan, Wei Cui, Ming Cai, Hui Fang

**Affiliations:** ^1^ Anhui Institute of Pediatric Research, Anhui Provincial Children’s Hospital, Hefei, China; ^2^ Anhui Province Key Laboratory of Medical Physics and Technology, Institute of Health and Medical Technology, Hefei Institutes of Physical Science, Chinese Academy of Sciences, Hefei, China; ^3^ Science Island Branch, Graduate School of University of Science and Technology of China, Hefei, China; ^4^ Department of Chinese Medicine, The First Affiliated Hospital of Anhui Medical University, Hefei, China; ^5^ Department of Pediatric Oncology Surgery, Anhui Provincial Children’s Hospital, Hefei, China; ^6^ Department of Hematology and Oncology, Anhui Provincial Children’s Hospital, Hefei, China; ^7^ Department of General Surgery, Anhui Provincial Children’s Hospital, Hefei, China; ^8^ Department of Scientific Research and Education, Anhui Provincial Children’s Hospital, Anhui Institute of Pediatric Research, Hefei, China; ^9^ Department of Pharmacy, The Second Affiliated Hospital of Anhui University of Chinese Medicine, Hefei, China

**Keywords:** hepatoblastoma, genetics, epigenetics, molecular mechanisms, interventions and treatments

## Abstract

Hepatoblastoma is a malignant embryonal tumor with multiple differentiation modes and is the clearest liver malignancy in children. However, little is known about genetic and epigenetic events in Hepatoblastoma. Increased research has recently demonstrated, unique genetic and epigenetic events in Hepatoblastoma, providing insights into its origin and precise treatment. Some genetic disorders and congenital factors are associated with the risk of Hepatoblastoma development, such as the Beckwith-Wiedemann syndrome, Familial Adenomatous polyposis, and Hemihypertrophy. Epigenetic modifications such as DNA modifications, histone modifications, and non-coding RNA regulation are also essential in the development of Hepatoblastoma. Herein, we reviewed genetic and epigenetic events in Hepatoblastoma, focusing on the relationship between these events and cancer susceptibility, tumor growth, and prognosis. By deciphering the genetic and epigenetic associations in Hepatoblastoma, tumor pathogenesis can be clarified, and guide the development of new anti-cancer drugs and prevention strategies.

## Introduction

Digestive system cancer represents one of the most conventional malignancies. It causes problems for clinicians and patients due to its high incidence and recurrence rates, limited drug options, and poor treatment outcomes. Hepatoblastoma is a malignant embryonal tumor with multiple differentiation modes and is the clearest liver malignancy in children (Calderwood et al., 1986). Its incidence occurs at a rate of 1.2–1.5 cases per million people annually. Most diagnoses are in children under 3 years and presentations above 4 years are extremely rare (Czauderna et al., 2014). Before the 1980s, the 5-year overall survival (OS) rate for children with Hepatoblastoma was only 36% (Hafberg et al., 2019). With the subsequent use of cisplatin chemotherapy and the development of a multidisciplinary treatment model, the 2022 cancer data show that the 5-year OS rate for children with Hepatoblastoma has reached an impressive 82%, with significantly improved prognoses. However, the path to treatment for advanced and refractory Hepatoblastoma remains challenging (Siegel et al., 2022). Additionally, the cause of Hepatoblastoma remains unknown. Meanwhile, studies have shown that some genetic disorders and congenital factors are associated with the risk of developing Hepatoblastoma, such as the Beckwith-Wiedemann syndrome (Mussa et al., 2019), Familial Adenomatous Polyposis (Giardiello et al., 1996), and Hemihypertrophy (Ahmad et al., 2016).

Although precision medicine has been increasingly incorporated into clinical practice and has enabled cancer prevention and treatment. However, the highly sensitive and specific genetic and epigenetic-related molecular markers currently used for clinical diagnosis, treatment, and prognosis are still unsatisfactory, especially as the molecular mechanisms involved in tumorigenesis, development, and treatment require further exploration and validation. Until now, the strong convergence shown by researchers towards genetics and epigenetics has contributed to further understanding Hepatoblastoma development.

Malignant tumors are the product of the interaction of environmental and host factors. The rapid proliferation, invasion, and metastasis of cells from carcinogenesis to tumor is a complex pathological process involving multiple factors and genes, accumulating through multiple change stages. An important feature of malignant tumor cells is the instability of their genome, which results in the creation of new mutated cells during cell division and the emergence of a population of mutated cells with different biological characteristics in the tumor cells (Mullard, 2020). Among these, genetic alterations and epigenetic modifications are also considered the main factors that regulate the development of cancer cells (Nam et al., 2021). On the other hand, genetic and epigenetic alterations are opposing concepts. Genetic alterations are based on changes in gene sequence leading to changes in gene expression levels, such as mutations, loss of gene heterozygosity, and microsatellite instability. Epigenetic modifications are based on non-genetic sequence changes such as DNA methylation and chromatin conformational changes leading to modifications in gene expression levels (Dawson and Kouzarides, 2012). The role of genetic factors in most tumorigenesis is to increase the body’s propensity to develop tumors and its susceptibility to carcinogenic factors. In contrast, abnormal epigenetic alterations work in multiple ways to activate single or multiple cellular pathways and genes, influencing the development of malignant tumors. Herein, we provided an overview of the genetic and epigenetic events in Hepatoblastoma (Table 1). Deciphering the genetic and epigenetic relationships in Hepatoblastoma will further elucidate its pathogenesis and guide the development of new anti-cancer drugs and prevention strategies.

**TABLE 1:** The keynotes of studies in each section of this review.

Section	Keynotes
Genetics and Hepatoblastoma	Beckwith-Wiedemann syndrome, Familial Adenomatous polyposis, Hemihypertrophy, Chromosomal aberrations, Chromosomal abnormalities, Wnt/β-catenin
Epigenetics and Hepatoblastoma	DNA methylation, Histone Modification, Non-coding RNA regulation (LncRNAs, miRNA)
Treatment associated with Hepatoblastoma	Surgical resection, Autologous or allogeneic liver transplantation, Chemotherapy and interventional therapy

## Genetics and hepatoblastoma

Cancer is caused by a mutation in one or more genesets, resulting in abnormal cell function, rapid and unrestricted growth, and the formation of malignant tumors. It is widely believed that the basic cause of cancer is genetic mutations. There are two possibilities for genetic mutations. One is inherited, passed from parent to child, increasing the risk of disease in the offspring. The other type is acquired later in life. Cancer is often easier to develop when genetic factors interact with environmental factors. If there are people in the patient’s immediate family who have Hepatoblastoma, this can increase the probability of the offspring developing the disease when influenced by the genetic factors involved. It is not the tumor itself, but the cancer susceptibility passed on from generation to generation. Hence, susceptible people are more likely to develop cancer in response to carcinogenic environmental factors than the general population.

### Genetically related disorders and hepatoblastoma

The Beckwith-Wiedemann syndrome is a cancer susceptibility syndrome caused by a defect in chromosome 11p15.5 and a congenital overgrowth disorder. Patients are usually at risk of overgrowth before birth and might develop neonatal hypoglycemia after birth, along with a giant tongue, enlarged internal organs, hemianopsia, and special creases and small indentations in the ears. This syndrome can predispose affected individuals to embryonic tumors such as nephroblastoma, rhabdomyosarcoma, adrenocortical carcinoma, neuroblastoma, and especially Hepatoblastoma (Derlin et al., 2018).

In a retrospective cohort study in Germany that evaluated cancer incidence and spectrum in children diagnosed with Belleville syndrome, 13 cancer cases were observed in the entire Beckwith-Wiedemann syndrome cohort, with Hepatoblastoma accounting for nearly 50% of the cases (*n* = 6) (Cöktü et al., 2020). (Sobel Naveh et al. (2022) conducted the first multidimensional study of samples collected from seven patients diagnosed with Beckwith-Wiedemann syndrome and Hepatoblastoma and showed that alterations in 11p15 drive Hepatoblastoma carcinogenesis by dysregulating chromatin organization. Steenman et al. (2000)further expanded their study by including solid childhood tumors associated with the Beckwith-Wiedemann syndrome, such as Wilms’ tumor, adrenocortical carcinoma, rhabdomyosarcoma, and Hepatoblastoma. They found that the development of these tumors shares a common genetic pathway involving chromosome 11. These studies were corroborated by Koufos et al. (1985)with the help of molecular probes in 1985. Children with Beckwith-Wiedemann syndrome have an increased potential for Hepatoblastoma progression *via* mutations at a locus on human chromosome 11. Zivot et al. (2020)demonstrated the close association of Hepatoblastoma with the Beckwith-Wiedemann syndrome based on magnetic resonance imaging, percutaneous puncture biopsy, and methylation testing. These authors suggested that all Hepatoblastoma patients should be considered for Beckwith-Wiedemann syndrome testing, even if they do not exhibit other diagnosable phenotypes. Additionally, Duffy et al. (2019)created predictive AFP values for preterm and non-preterm Beckwith-Wiedemann syndrome patients, establishing reference ranges for serum AFP at different ages in Beckwith-Wiedemann syndrome patients to help interpret and monitor the risk of Hepatoblastoma, as well as to better predict its occurrence in this population.

Familial Adenomatous polyposis (FAP) is an autosomal dominant disorder characterized by extensive adenomatous polyps throughout the colorectum. It is associated with APC gene mutation, an allele on the long arm of chromosome 5, 5q21-q22, and is at high risk of cancer (Vasen et al., 2008). Various other malignancies can occur in FAP, including Craniopharyngioma (Passos et al., 2020), Hepatoblastoma (Giardiello et al., 1996), Acute leukemia (Greenberg et al., 1981), and Thyroid and Pancreatic carcinoma (Giardiello et al., 1993). Achatz et al. clarified that multiple syndromes, including FAP, are associated with an increased incidence of gastrointestinal tumors and other cancer types by reviewing the literature on polyposis syndromes diagnosed in childhood and based on a workshop on childhood cancer susceptibility in October 2016; and even further recommended promoting cancer screening to them (Achatz et al., 2017). A possible non-random association between FAP and Hepatoblastoma has also been confirmed and might be associated with an increased risk. Numerous correlative studies have confirmed this to date. In a series of 93 Hepatoblastoma patients, eight suggested a family history of FAP (Hirschman et al., 2005). In 1987, identical twin boys diagnosed with Hepatoblastoma had polyps in the colon of their mother and grandmother, and their Hepatoblastoma was possibly associated with FAP (Riikonen et al., 1990). These observations reinforce the possible link between the two conditions. Trobaugh-Lotrario et al. (2018)reviewed the Hepatoblastoma cases in FAP patients reported in the literature. Of the 49 patients with available data, 35 were diagnosed with FAP before they were diagnosed with Hepatoblastoma. They highlighted the need for earlier identification and screening of infants in the FAP family for Hepatoblastoma. Multiple synchronous tumors in infancy also require earlier identification and screening. In one reported case, a 7-month-old male infant with concurrent Beckwith-Wiedemann syndrome, FAP, and Li-Fraumeni syndrome, and an autopsy showing Hepatoblastoma, further indicated a unique association between multiple synchronous tumors and genetics (Ozawa et al., 2016). A maternally inherited APC mutation was confirmed in another surviving child diagnosed with metastatic Hepatoblastoma (Liu et al., 2021a). The association of Hepatoblastoma in FAP families can occur through affected relatives. Gupta et al. noted that the risk of Hepatoblastoma in FAP individuals can increase to terrifying 750 to 7500 times (Gupta et al., 2013). Since there is a clear correlation between Hepatoblastoma and FAP, any treatment for FAP might be a possible therapeutic target for Hepatoblastoma. A Randomized Controlled Trial (RCT) demonstrated the efficacy of low-dose aspirin as an alternative method of preventing FAP-related colorectal cancer (UMIN000018736) (Ishikawa et al., 2021). It has also been demonstrated that Metformin (NCT01725490) (Park et al., 2021) and Curcumin (NCT00641147) (Cruz-Correa et al., 2018) are ineffective for FAP treatment. There is still a need to screen children affected by FAP for serum methemoglobin levels and abdominal computed tomography (CT) scans.

Hemihypertrophy is a progressive asymmetry of the limbs and trunk on one-half of the body. It mainly occurs in infants and children, and its etiology and pathogenesis are unknown. The diagnosis of Hemihypertrophy is based on clinical presentation and relevant laboratory tests, which also requires an experienced clinical geneticist to rule out other limb asymmetry causes, such as neurological or skeletal abnormalities or tumors. No specific genetic changes cause Hemihypertrophy, and the number, location, and function of the genes involved are unknown. Furthermore, it is impossible to predict the risk of Hemihypertrophy for tumor development with current molecular techniques. Hence, routine screening for tumors is required in all children.

**FIGURE 1:**
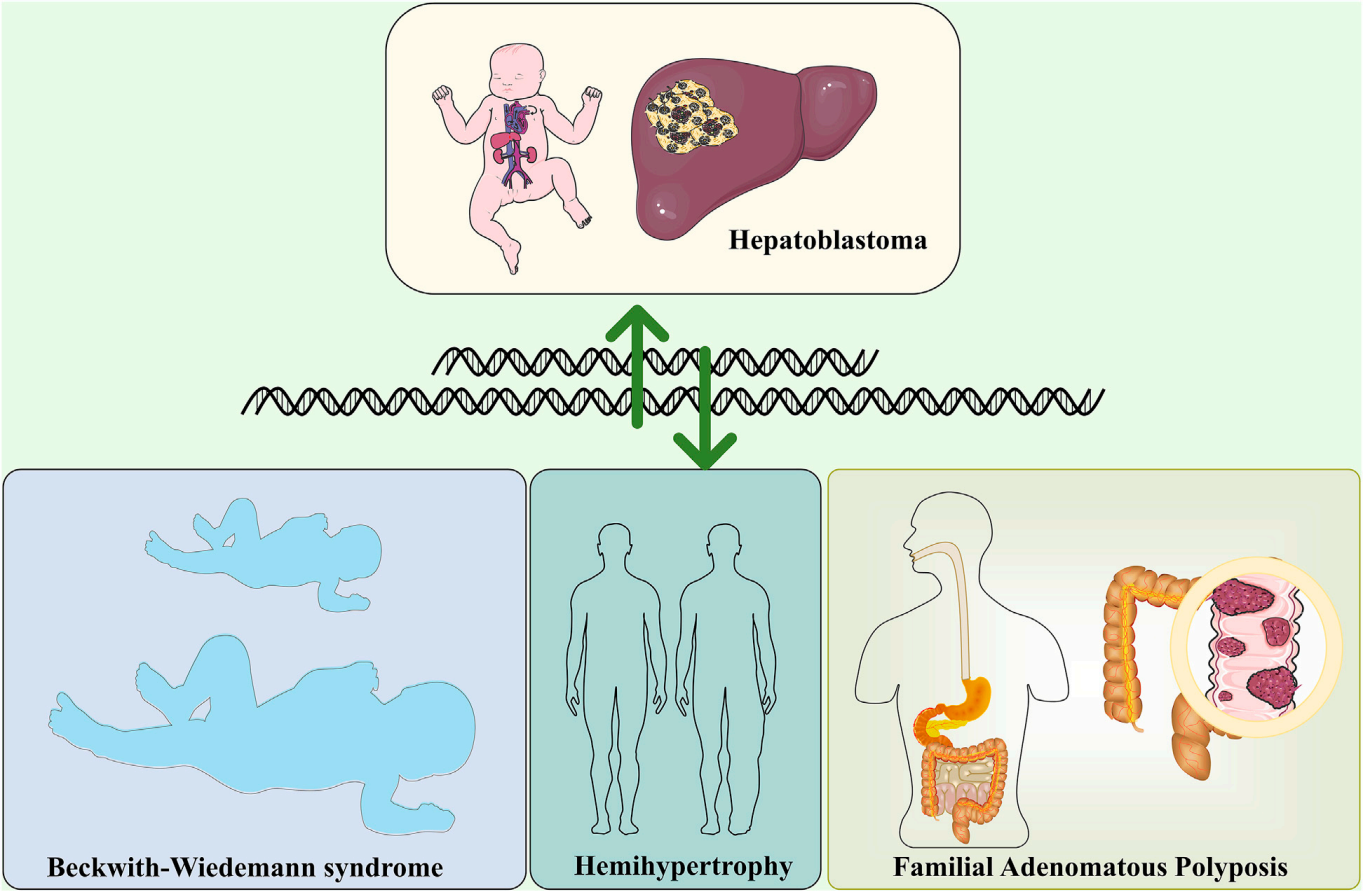
Hepatoblastoma and genetically related disorders, including Beckwith-Wiedemann syndrome, Familial Adenomatous polyposis and Hemihypertrophy.

Hemihypertrophy is associated with an increased risk of embryonic tumors, mainly Wilms’ and Hepatoblastoma. In a retrospective survey, Dempsey et al. found that Hemihypertrophy had a significantly lower risk of concomitant tumors than other syndromes, with an incidence of only 1.2% (Dempsey-Robertson et al., 2012), similar to an earlier study. In the nine Hemihypertrophy cases investigated, only one case developed Hepatoblastoma (Geormăneanu et al., 1983). There is often a partial overlap in clinical management between Hemihypertrophy and Beckwith-Wiedemann syndrome. Most Beckwith-Wiedemann syndrome patients have a defect in chromosome 11p15.5. In contrast, Hemihypertrophy patients do not have an identifiable cause. Therefore, Clericuzio and Martin. (2009)recommended that all Hemihypertrophy patients be screened for tumors. The association between Hemihypertrophy and Hepatoblastoma remains a concern for many scholars to find an early breakthrough in Hepatoblastoma treatment (Rattan et al., 1995). Besides the above Hepatoblastoma-related genetic disorders, Simpson-Golabi-Behmel syndrome, trisomy 18 (Nussbaumer and Benesch, 2022), and ARID1A Coffin-Siris syndrome (Cárcamo et al., 2022) are also associated with Hepatoblastoma (Figure 1).

### Cell genetics and hepatoblastoma

Chromosomes are mainly composed of DNA and proteins and act as carriers of genetic information. Chromosomes have a species specificity and vary in number, size and morphology depending on the organism, cell type, and development stage. The most notable are chromosomal aberrations, which are changes in the number and structure of a cell’s normal chromosomes. Some chromosomal aberrations are a prominent cause of human genetic disorders. For example, three copies of chromosome 21 exist in Down’s syndrome (Roizen and Patterson, 2003). Additionally, chromosomal translocations might increase the chances of their offspring developing the disease (Nambiar and Raghavan, 2013). Chromosomal aberrations have an important place in oncology research. In a follow-up survey of almost 30 years, 2396 healthy Hungarians were tested cytogenetically. Interestingly, smoking intensity was a predictor of chromosomal aberrations rather than duration. It was also confirmed that cancer incidence was associated with chromosomal aberrations (Farkas et al., 2021). Another sizeable, pooled cohort study showed that the frequency of chromosomal aberrations was significantly associated with cancer risk and predicted the risk of developing cancer (Bonassi et al., 2008). Chromosomal instability and aneuploidy are also hallmarks of cancer cells and are often associated with aggressiveness and poor prognosis (Chiarle, 2021).

There are also multiple chromosomal abnormalities found in Hepatoblastoma. Previous studies have highlighted the relevance of numerical and structural abnormalities of chromosomes 1q, 4q, 2, 8, and 20 in Hepatoblastoma development. Nagata et al. reported a Hepatoblastoma case in which chromosomal translocation was unbalanced, and 4q35 was the only chromosomal abnormality of a unique type (Nagata et al., 2005). In a genome-wide analysis of chromosomal aberrations in Hepatoblastoma based on high-density single nucleotide polymorphism genotyping microarrays, 17 Hepatoblastoma samples presented chromosomal aberrations, observable in approximately 88% of the samples and increased in chromosomes 1q, 2/2q, 8, 17q, and 20. Chromosomes 4q and 11q were frequently lost. Suzuki et al. further added to the above studies (Suzuki et al., 2008). Furthermore, chromosome 2 and 20 trisomies were the most consistent changes in these studies, and their frequent occurrence in Hepatoblastoma suggests their pathogenic significance (Bardi et al., 1992; Yeh et al., 2000). This was also corroborated by a comparative study by Sumazin et al. They used a diagnostic algorithm to identify molecular signatures of Hepatoblastoma and Hepatocellular carcinoma and observed recurrent large-scale chromosomal gains, including gains in chromosome arms 2q, 6p, and 20p, all associated with poor prognosis (Sumazin et al., 2022).

**FIGURE 2:**
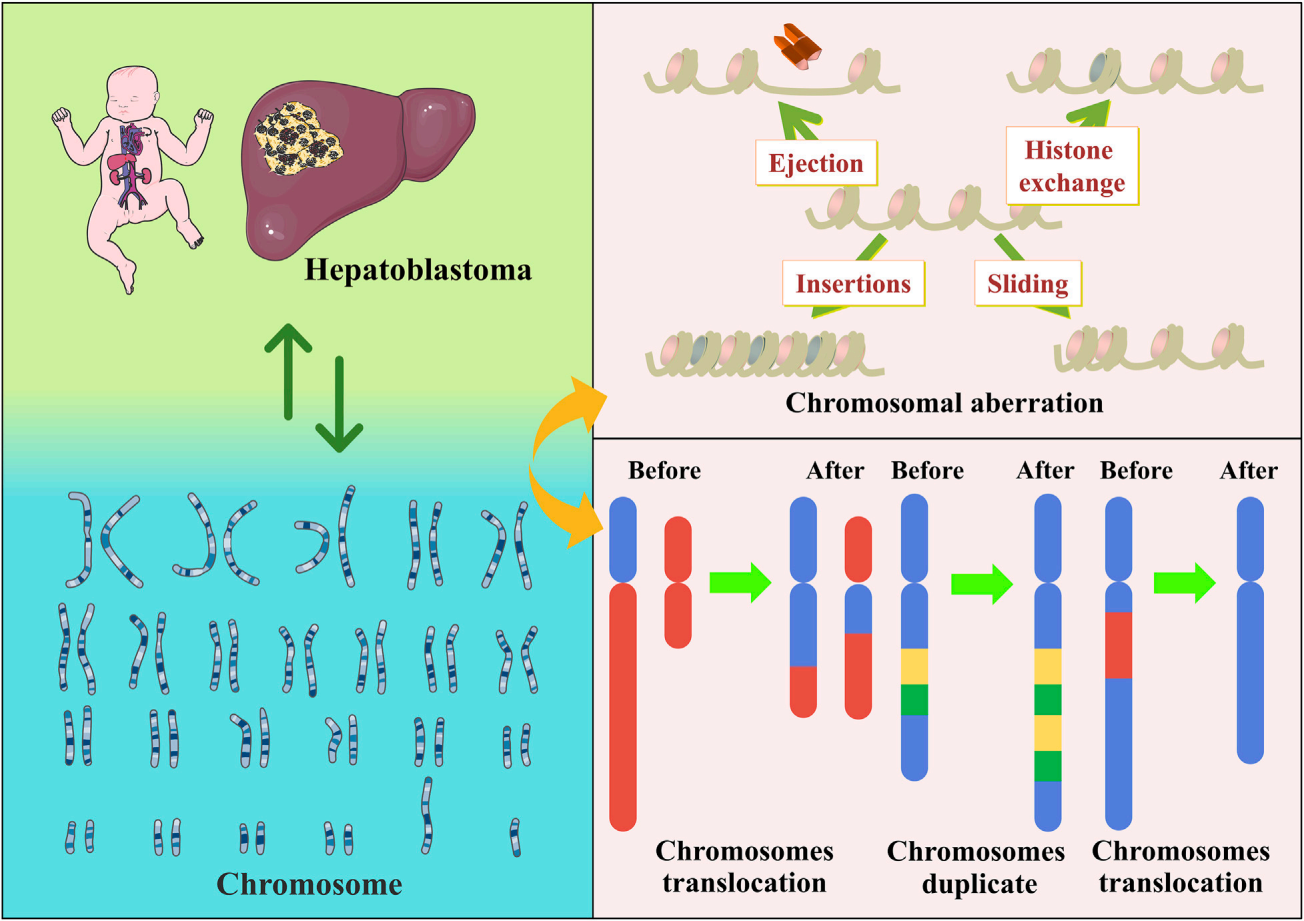
Hepatoblastoma has a clear association with chromosomes, as exemplified by chromosomal aberrations.

Furthermore, sequencing analysis of human Hepatoblastoma specimens revealed a crucial role of the Wnt/β-catenin protein pathway (Zhang et al., 2021). The Wnt/β-catenin signaling pathway is a conserved signaling axis involved in various pathophysiological processes, including cell proliferation, apoptosis, invasion, and migration. Targeting the Wnt/β-catenin signaling pathway can inhibit self-renewal, cell proliferation, and differentiation of various cancer stem cells (Huang et al., 2020). Some studies have also linked the Wnt/β-catenin gene to chromosomal aberrations. Fukuzawa detected mutations or accumulation of β-catenin proteins in both cases of Hepatoblastoma with Beckwith-Wiedemann syndrome, suggesting that Wnt signaling activation can be a subsequent event involving Beckwith-Wiedemann syndrome-associated Hepatoblastoma (Fukuzawa et al., 2003). Sha et al. (2019) conducted a review based on a large body of evidence from preclinical and clinical studies suggesting that Wnt/β-catenin protein is a potential therapeutic target for Hepatoblastoma. Its mechanisms involve somatic mutations in exon three of the β-catenin protein gene, which is activated in Hepatoblastoma. This was also confirmed by Wang et al. (2019). There are also common signaling pathways involved in Hepatoblastoma development, such as PI3K/Akt/mTOR, MAPK, and p53. Cui et al. (2019)found that miR-193a-5p directly targets DPEP1 and participates in Hepatoblastoma progression by regulating the expression of the PI3K/Akt/mTOR signaling pathway. The lncRNA MIR205HG has also been reported to accelerate cell proliferation, migration, and invasion in Hepatoblastoma by activating the MAPK and PI3K/AKT signaling pathways (Zhang et al., 2022). Yamamoto et al. (2007)suggested that reduced expression of the tumor suppressor p53 could confer resistance to oxidative stress in Hepatoblastoma, partly contributing to its development. Additionally, other related pathways involved include NF-κB, JAK/STAT, and TGFβ/SMAD (Buenemann et al., 2001; Nagai et al., 2003; Wang et al., 2022) (Figure 2).

## Epigenetics and hepatoblastoma

Epigenetics was initially proposed by Conrad H. Waddington and became a research hotspot with the resolution of the DNA double helix structure and the rapid development of biochemical techniques (Peixoto et al., 2020). It typically refers to heritable changes resulting from non-genetic sequence alterations, such as DNA methylation, histone modifications, and non-coding RNA regulation of chromatin, associated with the occurrence and progression of many prevalent diseases, including cancer (Flavahan et al., 2017; Harvey et al., 2018).

### DNA methylation

**FIGURE 3:**
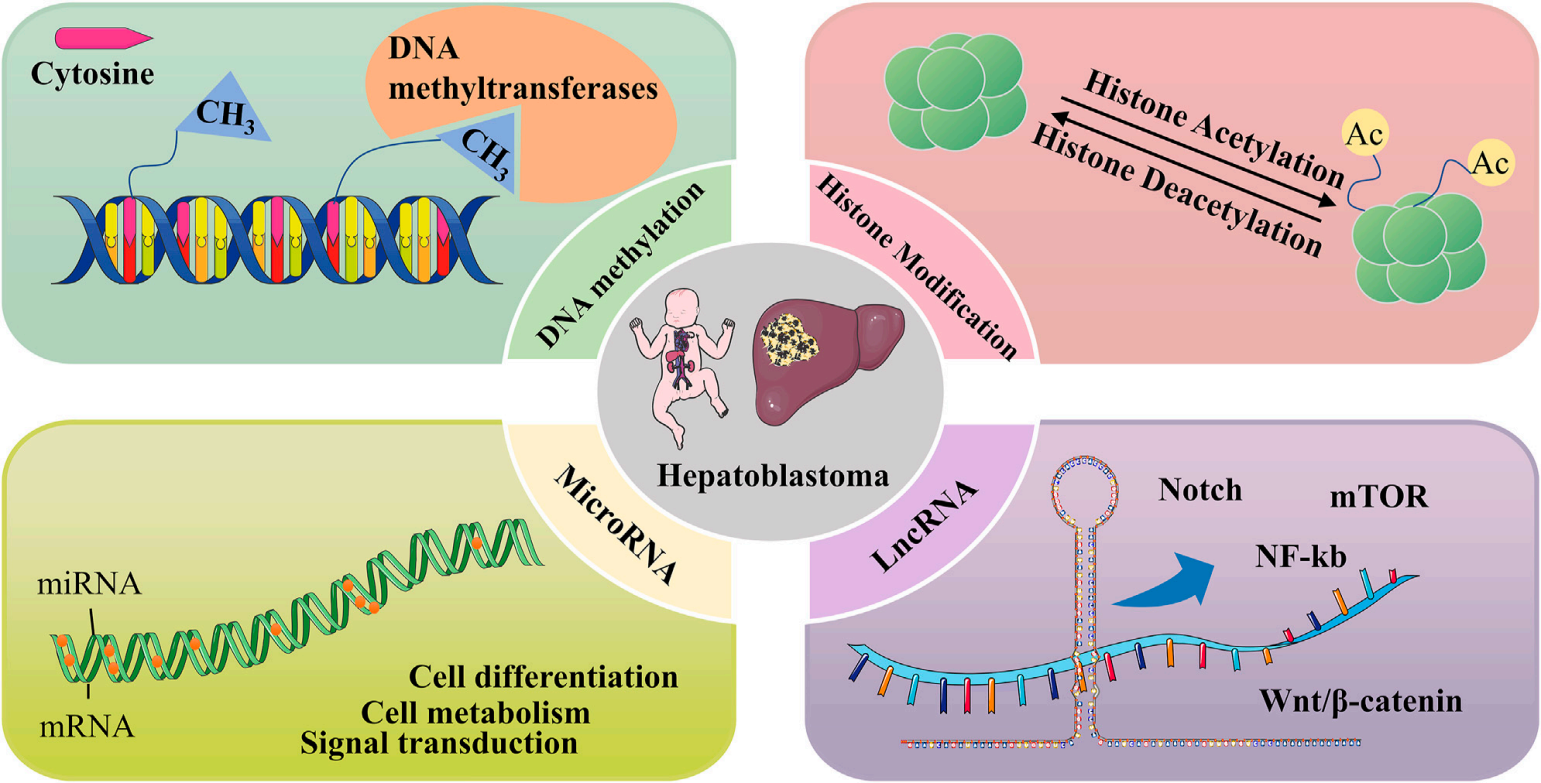
Epigenetic mechanisms involved in Hepatoblastoma, including DNA methylation, histone modifications, and regulation of non-coding RNAs.

DNA methylation regulates gene expression at an epigenetic level. The covalent addition of methyl usually occurs to 5-carbon cytosine nucleotides to produce 5-methylcytosine, a reaction usually established and maintained by DNA methyltransferases (von Känel and Huber, 2013). DNA methylation regulates several processes, such as embryonic development, transcription, chromatin structure, and chromosome stability, and its defects can lead to various diseases (genomic instability) (Meng et al., 2015). It is also an epigenetic regulator of gene expression and induces the silencing of various oncogenes, such as cell cycle regulators, pro-differentiation factors, DNA repair proteins, and anti-apoptotic factors (Esteller, 2008; Fukushige and Horii, 2013). The CpG-rich regions are known as CpG islands, which are usually unmethylated. Additionally, most of the human genome is methylated (Lister et al., 2009). Numerous studies have shown that many cancers are associated with promoter-specific hypermethylated CpG islands and concomitant gene silencing (Kulis and Esteller, 2010; Morgan et al., 2018). Local hypermethylation of tumor suppressor promoters in cancer can also disrupt cancer-related cellular pathways, such as DNA repair, cell cycle, P53 network, apoptosis, Ras signaling, and Wnt signaling (Pan et al., 2021). Besides the hypermethylation of CpG islands described above, DNA hypomethylation can also induce tumorigenesis (Klutstein et al., 2016). For example in Figure 3, in Hepatoblastoma, demethylation of the igf2 P3 region causes upregulation of promoter activity, while hypermethylation of h19 DNA leads to downregulation. This gene expression alteration due to aberrant methylation might be important in Hepatoblastoma development (Li et al., 1998).

Altered DNA methylation patterns are important in Hepatoblastoma, where genes show global hypomethylation changes, specific tumor suppressor genes show hypermethylation, and significantly higher methylation levels in Hepatoblastoma tissue than in non-tumor tissues (Cui et al., 2016; Shen et al., 2020). RASSF5 is a member of the RAS-associated domain family and participates in cell growth regulation, and is inactivated by promoter hypermethylation in various cancers, including Hepatoblastoma (Suryaraja et al., 2013). Liu et al. (2018a)measured the RASSF5 methylation status in nine pairs of Hepatoblastoma and normal liver tissues, which showed that all five CpG loci in the RASSF5 gene region were significantly more methylated in Hepatoblastoma tissues than in normal liver tissues. Similarly, Sakamoto et al. (2010) performed methylation tests on several paraffin-embedded Hepatoblastoma and normal liver samples showing high levels of tumor-specific DNA hypermethylation in the promoter regions of APC, CDH1, MT1G, RASSF1A, and SOCS1, with MT1G hypermethylation showing a slight association with poor prognosis in Hepatoblastoma patients.

Several studies have shown that methylation genes are associated with the Hepatoblastoma prognosis as a key predictor. Nagae et al. (2021) performed methylation analysis of CpG island promoters and liver enhancers in 154 Hepatoblastomas, which indicated that enhancers rich in ASCL2-regulated transcription factors exhibited hypomethylation. Promoter hypermethylated isoforms were useful for assessing prognostic risk in Hepatoblastoma, in which DLX6-AS1 hypermethylation was predicted as an important molecular marker. Additionally, Honda et al. detected nine methylation genes in Hepatoblastoma tumor samples. GPR180, MST1R, OCIAD2, and PARP6 were associated with tumor suppressors, and their hypermethylation suggested a poor tumor prognosis. There was also an association between methylation status and the onset age of Hepatoblastoma patients (Honda et al., 2016). RASSF1A is a tumor suppressor in cell signaling and acts as a hub in signal transduction. DNA hypermethylation causes the silencing of RASSF1A expression in various tumors (García-Gutiérrez et al., 2020). In a related study, the methylation status of the RASSF1A promoter region was examined using bisulfite pyrosequencing, and the results showed that RASSF1A methylation was present in several patients who developed recurrence or distant metastases after surgery, leading to a strong correlation between RASSF1A methylation and tumor prognosis (Honda et al., 2013). Honda et al. (2008) evaluated 33 patients with stage 3B or four and showed that patients with methylated tumors had a lower OS rate than patients with unmethylated tumors.

### Histone modification

Histones are the spool of twisted DNA and form the nucleosome. Two molecules of each of the four core histones, H2A, H2B, H3, and H4, form the core of the nucleosome (Audia and Campbell, 2016). Many specific residues in the tails of these histones can undergo post-translational modifications that affect DNA-associated processes, including chromatin structure and transcription (Lawrence et al., 2016). Post-translational modification of histones is a reversible process that includes various modifications such as methylation, acetylation, ubiquitination, and acylation. Abnormal regulation of post-translational modifications is associated with cancer development. For example, inappropriate activation and inactivation of oncogenes in cancer can be caused by the dysregulation of histone modifications (Park and Han, 2019; Borkiewicz, 2021). Additionally, in most cancers, epigenetic and metabolic states can interact, with altered metabolism causing abnormal epigenetic regulation, which can even drive immune escape and promote adverse tumor progression (Thakur and Chen, 2019; Sun et al., 2022). There are relatively few Hepatoblastoma studies on histone modification and it is a relatively blank area.

Acetylation of lysine residues in histones is a common histone modification regulated by two enzymes with opposite effects, histone acetyltransferases (HATs) and histone deacetylases (HDACs). Resveratrol has an antiproliferative effect on the Hepatoblastoma cell line HepG2, and this effect is associated with its specific inhibition of HDACs, causing histone hyperacetylation (Venturelli et al., 2013). Liu et al. found that quercetin can promote the histone H3K9 deacetylation by upregulating SIRT6, thus affecting the subordinate pathway to inhibit malignant phenotypes, such as proliferation and metastasis of Hepatoblastoma cells (Liu et al., 2021b). Tumor necrosis factor-related apoptosis-inducing ligand (TRAIL) is a potential antitumor agent. For example, combining a histone deacetylase inhibitor with TRAIL can improve the resistance of Hepatoblastoma cell lines to TRAIL, which is friendly to normal cells (Dzieran et al., 2008).

### Non-coding RNA regulation

Long non-coding RNAs (lncRNAs) are non-coding RNAs with more than 200 nucleotides that do not normally encode proteins but are involved in various cellular processes and pathways. Their aberrant expression has been shown to play an important role in many cancer-related signaling molecules/pathways, such as Notch, mTOR, NF-kb, and the Wnt/β-catenin pathway, and can affect various malignant phenotypes in cancer cells (Chi et al., 2019; Chen et al., 2020). Since lncRNAs are involved in various cellular processes and are associated with multiple pathways, increasing research suggests that mutations or dysregulation of lncRNAs can lead to abnormal gene expression and contribute to tumorigenesis and progression (Sharma et al., 2020). In a genome-wide analysis of lncRNA expression in Hepatoblastoma tissues, 2736 lncRNAs were differentially expressed. Among them, 1757 lncRNAs were upregulated, and 979 lncRNAs were downregulated compared to normal tissues. Additionally, about a quarter of the 420 matched lncRNA-mRNAs in tumor tissue were differentially expressed lncRNAs (Dong et al., 2014).

Increasing studies have demonstrated that multiple lncRNAs can play a regulatory role in Hepatoblastoma development. Zhu et al. (2021) found that the expression of NBR2 was upregulated under glucose starvation. The lncRNAs NBR2 and TCF7 are competitively bound to miR-22, and the inhibitory effect of miR-22 on TCF7 decreases with reduced binding, thereby exacerbating the malignancy of Hepatoblastoma cells. The lncRNA NBR2 could serve as a potential target to suppress the malignant phenotype of Hepatoblastoma. Moreover, the lncRNA TUG1 was significantly upregulated in Hepatoblastoma specimens and cells, and TUG1knockdown inhibited tumor growth *in vivo* and the proliferation and migration of Hepatoblastoma cells *in vitro*, suggesting that regulation of the lncRNA TUG1 is also one of the targets for Hepatoblastoma treatment (Dong et al., 2016). Additionally, lncRNA not only serves as a therapeutic target but also affects various malignant phenotypes of Hepatoblastoma by regulating signaling pathways. The lncRNA MIR205HG is significantly overexpressed in Hepatoblastoma and competitively binds to miRNA-514a-5p to activate the mitogen-activated protein kinase signaling pathway. It also activates the PI3K/AKT signaling pathway, and lncRNA MIR205HG promotes the proliferation, migration, and invasion of Hepatoblastoma by activating both pathways (Zhang et al., 2022). The lncRNA OIP5-AS1 can activate β-catenin signaling by promoting binding between PTBP1 and β-catenin, thereby promoting Hepatoblastoma proliferation and silencing. Thus, OIP5-AS1 might be a potential target to counteract Hepatoblastoma (Jiang et al., 2022). Also, lncRNAs have been associated with tumor risk. H19 is a highly conserved lncRNA transcript known as lncRNA-H19. It is associated with the development of various tumors and plays an important role in epigenetic, transcriptional, and post-transcriptional regulation (Lecerf et al., 2019). In a survey of 213 Hepatoblastoma patients, three H19 polymorphisms, rs2839698, rs3024270, and rs217727, were associated with susceptibility to Hepatoblastoma. The rs2839698 and rs3024270 polymorphisms were associated with a significantly increased risk of Hepatoblastoma, while rs217727 was associated with a significantly decreased risk (Tan et al., 2021).

Furthermore, microRNAs (miRNAs) are short-stranded non-coding RNAs that can influence many biological processes, such as cell differentiation, cell metabolism, and signal transduction, by regulating gene expression. Their dysregulation is associated with various diseases, including cancer (Iorio and Croce, 2012). In cancer, miRNAs can influence tumor immune processes with miRNA sponges (He et al., 2020). Moreover, miRNAs regulate not only genes but also multiple signaling pathways. Each miRNA can regulate multiple target genes and influence the activity of signaling pathways, corresponding to the fact that many different miRNAs regulate a particular gene and that a gene might have more than half a hundred miRNA binding sites (Okugawa et al., 2015). In a comprehensive genomic analysis of the Hepatoblastoma miRNA-mRNA interaction network, clusters of differentially expressed miRNAs were detected between fetal-type tumors, embryonic-type tumors, and corresponding normal liver groups, with 33 upregulated and 12 downregulated hub miRNAs in the intersection of these two clusters (Chen et al., 2021).

Several studies have shown that miRNAs can play an important role in regulating Hepatoblastoma status. By analyzing the expression of miR-17, miR-146a, miR-302days, and miR-19b in 22 Hepatoblastoma tumor samples and 10 surrounding normal liver samples, Ecevit et al. demonstrated that miRNA-17 expression levels were lower, and miR-19b expression levels were higher in Hepatoblastoma samples compared to surrounding normal liver samples. This lower miRNA-17 expression could also predict prognosis in Hepatoblastoma patients (Ecevit et al., 2019). Another miRNA, miR-492, was studied by Frowein et al. The non-kinase transmembrane glycoprotein CD44 is a target of miR-492, which can act directly on the CD44 target to affect the ability of Hepatoblastoma cells to proliferate and metastasize. Besides, miR-492 can be used as a biomarker for Hepatoblastoma prognosis, and its high expression can promote Hepatoblastoma malignant phenotypes (von Frowein et al., 2018; Chen et al., 2018). Cui et al. showed that miR-186 overexpression significantly suppressed the malignant phenotype of Hepatoblastoma cells, and methyltransferase-like 3 (METTL3) inhibited this miR-186 overexpression. The miR-186/METTL3 axis promoted the proliferation and migration of Hepatoblastoma cells through the Wnt/β-catenin signaling pathway, which might be a target for the treatment of Hepatoblastoma (Cui et al., 2020). Liu et al. identified 869 differentially expressed circular RNAs (circRNAs) between Hepatoblastoma samples and corresponding normal liver samples. These circRNAs can fine-tune gene expression and influence tumor development. For example, circ_0015756 is a sponge for miR-1250–3p and was significantly upregulated, while its silencing suppressed the proliferation and metastatic capacity of Hepatoblastoma cells (Liu et al., 2018b; Kristensen et al., 2022).

## Treatment associated with hepatoblastoma

With the development of cross-disciplinary disciplines such as medical imaging and molecular biology, Hepatoblastoma treatment has advanced, including the discovery of targeted pathways and clinical trials. There is now a consensus on the traditional treatment options for Hepatoblastoma, including surgical resection, autologous or allogeneic liver transplantation, chemotherapy, and interventional therapy. Sunil et al. (2018) analyzed Hepatoblastoma patients who underwent hepatectomy between 2000 and 2013. They analyzed their survival outcomes and concluded that patients who underwent surgery had a well-controlled disease and could have long-term survival. Liver transplantation is the only effective treatment for end-stage liver disease. (Filin et al. (2020) followed 19 Hepatoblastoma patients who underwent liver transplantation over a long time and found that their 4-year survival rate was 68%. Liver transplantation has shown a favorable outcome in Hepatoblastoma, as demonstrated by another retrospective analysis. After 20 years of prolonged follow-up, liver transplantation showed a favorable outcome in cases of unresectable Hepatoblastoma, and the number of perioperative complications was acceptable (Moosburner et al., 2021). Additionally, interventions and chemotherapy play an important role. Transcatheter arterial chemoembolization (TACE) has been considered a unique and remarkable tool for local Hepatoblastoma control, which can maximize the antitumor effect of the drug with good efficacy, rapid efficacy, and minimal side effects (Hishiki, 2013).

Hepatoblastoma is one of the clinical challenges of childhood disease because it is common and often unresectable. Vogl et al. (2006) recognized the benefits of the TACE technique and showed that its combination with other treatments could be an alternative to surgery and offer new hope for patients with unresectable tumors. In a study of high-intensity focused ultrasound (HIFU) combined with TACE for Hepatoblastoma, 12 unresectable Hepatoblastoma patients presented survival rates at 1 and 2 years of 91.7 and 83.3%, respectively. The combination of HIFU and TACE is a safe and promising approach with a low incidence of serious complications. As a minimally invasive approach, it might offer a new local treatment for unresectable Hepatoblastoma patients (Wang et al., 2014). Although the efficacy of TACE is positive, it also has limitations, such as incomplete embolization leading to treatment failure and inappropriateness in cases where the patient is in poor health and intolerant to this treatment.

Hepatoblastoma is the most common malignant liver tumor in children. Recently, its prognosis has been greatly improved with the development of optimized chemotherapy schemes, improved surgical approaches, and the maturation of liver transplantation techniques. Moreover, many treatments previously used for adult liver tumors, such as oncologic interventional chemotherapy and ultrasound focusing, have been gradually introduced in children (Wang et al., 2014). Additionally, gene-targeted therapy, tumor-induced differentiation therapy, and immunotherapy have also been developed in basic experiments and clinics (Li et al., 2017).

In refractory Hepatoblastoma, when conventional surgery and radiotherapy fail, target-specific inhibitors might also be considered for precision therapy, which is essential for developing relevant treatment protocols and assessing treatment and prognostic outcomes. Currently, the classical targeting signaling pathways for Hepatoblastoma include PI3K/Akt and Wnt/β-catenin. Compared to chemotherapeutic drugs with non-specific targets, targeted drugs are ideal and effective treatments because they are highly specific and kill fewer normal cells. Some targeting factors, such as PI3K, mTOR, and Wnt, are also being explored. These targeting factors can selectively interfere with targeted pathways such as tumor growth, development, and vascular development.

**FIGURE 4:**
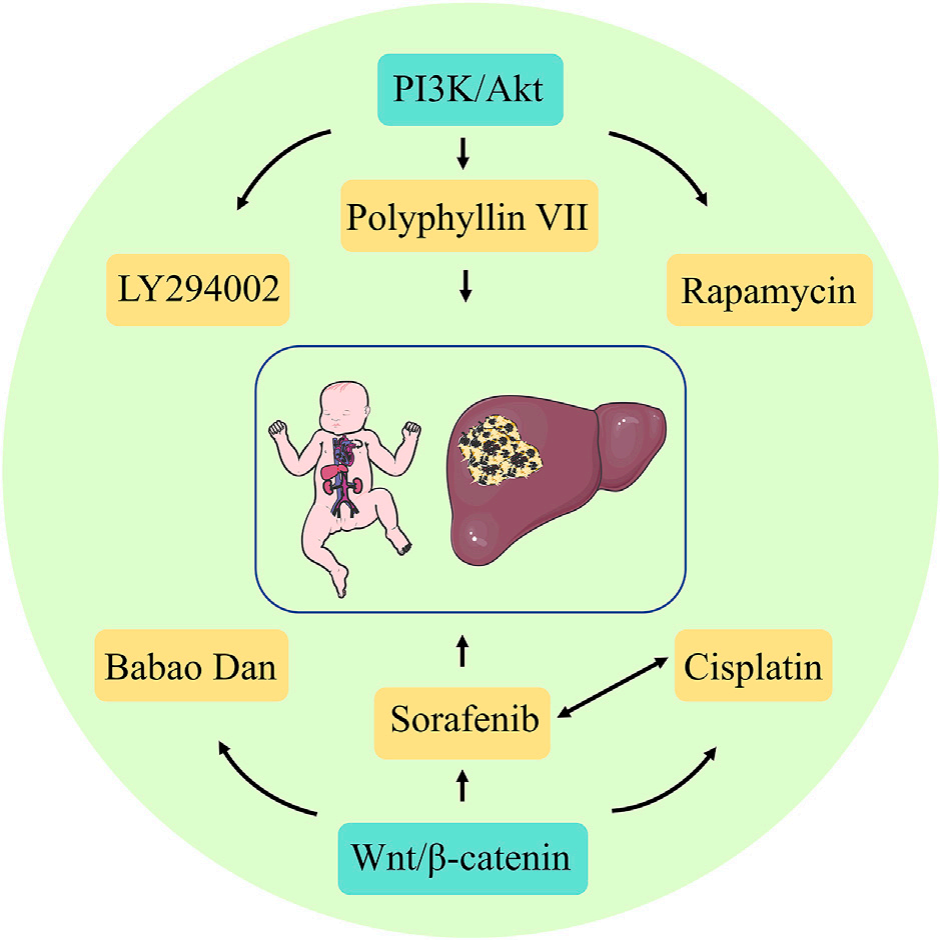
Major targeted signaling pathways and targeted drugs involved in Hepatoblastoma.

Furthermore, PI3K is a dimer consisting of the regulatory subunit p85 and the catalytic subunit p110. When PI3K binds to the growth factor receptor, it can alter Akt protein structure and activate it, activating or inhibiting the activity of various downstream substrates by phosphorylation, thereby regulating cell proliferation, differentiation, apoptosis, and migration phenotypes. PI3K and Akt are specifically highly expressed in Hepatoblastoma. Thus, the PI3K/Akt pathway is involved in the development of Hepatoblastoma. When the proteins involved in this signaling pathway are directly targeted and inhibited, Hepatoblastoma proliferation and metastasis can be effectively controlled (Zhang et al., 2021). Classical targeted drugs include the PI3K inhibitor LY294002, mTOR inhibitor rapamycin, and others. Hartmann et al. (2009) used LY294002 to treat a Hepatoblastoma cell line and found a positive reversal effect, which significantly reduced the growth of Hepatoblastoma cells by increasing apoptosis and decreasing cell proliferation. Wagner et al. (2012) used an oral dose of rapamycin of 5 mg/kg/day for 3 weeks to treat mice with subcutaneous Huh6 xenograft tumors and observed the therapeutic effect. They found that rapamycin significantly reduced tumor growth in mice, as well as reduced tumor-specific AFP serum levels. The herb Polyphyllin VII has strong anticancer activity in various cancer types and induces autophagy and apoptosis in HepG2 cells by inhibiting the PI3K, AKT, and mTOR phosphorylation. Thus, it can act as an mTOR inhibitor (Zhang et al., 2016). The Wnt/β-catenin protein pathway has emerged as a research priority as one of the more well-defined Hepatoblastoma initiation mechanisms. In healthy livers, β-catenins are inactive due to the absence of extracellular Wnt ligands. In contrast, in malignant tumors, the presence of Wnt ligands due to functional loss or mutations in genes, such as APC, increases the susceptibility to Hepatoblastoma development (Perugorria et al., 2019). β-Catenins are important targets for Hepatoblastoma therapy. Extensive studies have confirmed that inhibition of Wnt/β-catenin protein signaling in Hepatoblastoma can lead to surprising therapeutic effects, and many approaches to inhibit this signaling have been reported, including siRNA, miRNA, and pharmacological agents (Sha et al., 2019). Xie et al. (2021) elucidated the value of traditional Chinese medicine (TCM) for Hepatoblastoma. They showed that Babao Dan dramatically attenuated the activation of the Wnt/β-catenin protein pathway in Hepatoblastoma and significantly reduced the expression of Wnt target genes and cancer stem cells. Furthermore, sorafenib, a multi-targeted oral drug for treating tumors, selectively targets the receptors of certain proteins and might act as a molecular switch during tumor growth. The application of sorafenib in Hepatoblastoma treatment effectively inhibits cell viability, tumor progression, and angiogenesis (Eicher et al., 2012). Its combination with cisplatin also significantly reduces Hepatoblastoma cell viability and is a promising treatment option for high-risk or recurrent Hepatoblastoma (Eicher et al., 2013). Immunotherapy can be used as a branch of targeted therapy, usually targeting the body’s immune regulatory processes against tumors and correcting immune escape occurrence, with a high generalization degree. For refractory or resistant Hepatoblastoma after surgery and chemotherapy, immunotherapy might induce an immune response against the tumor cells, thereby inhibiting the Hepatoblastoma development (Figure 4).

Although there are several commonly used anti-tumor drugs, such as sorafenib and cisplatin, new drugs are still being developed. However, there must be a rational treatment schedule, including administration timing, selection and combination of drugs, administration sequence, dosage, and treatment course and interval, to achieve a comprehensive, rational, and effective choice of combination chemotherapy schedule. Hence, many clinical trials have been conducted to find the optimal treatment for Hepatoblastoma. These trials are presented in Table 2.

**TABLE 2:** Clinical trials for hepatoblastoma.

NCT number	Drug	Condition	Conclusion	Ref
NCT00077389	Cisplatin	High-risk Hepatoblastoma	Feasible and effective	Kristensen et al. (2022)
NCT00980460	Cisplatin, fluorouracil, vincristine	Hepatoblastoma with surgical resection	Minimal post-operative chemotherapy ensures control of patient’s condition	Sunil et al. (2018)
NCT00003912	Cisplatin *versus* cisplatin plus doxorubicin	Standard-risk Hepatoblastoma	Similar rates of complete resection and survival	Filin et al. (2020)
NCT00428272	Lexatumumab	Hepatoblastoma	Showed a dramatic biomarker response	Moosburner et al. (2021)
NCT00929903	Pazopanib	Hepatoblastoma	Achieved a partial response	Hishiki, (2013)
NCT00652132	Sodium Thiosulfate, Cisplatin	Hepatoblastoma	Sodium Thiosulfate for Protection from Cisplatin-Induced Hearing Loss	Vogl et al. (2006)
NCT00716976	Sodium Thiosulfate	Hepatoblastoma	Protects against cisplatin-induced hearing loss	Wang et al. (2014)
NCT01331135	Sirolimus, metronomic therapy (CHOAnome)	Hepatoblastoma	Well tolerated	Li et al. (2017)

## Conclusion and perspectives

Unraveling the genetic changes and epigenetics involved in cancer pathogenesis is challenging and remains a necessary task. Despite excluding the worldwide impact of COVID-19, the latest cancer data show that the number of cancer deaths continues to rise annually (Siegel et al., 2022). With a deeper understanding of genetic changes and epigenetics, new clues have been provided to the molecular mechanisms of disease. Nevertheless, there is still an urgent need to translate these findings into the clinic for application in diagnosis, prognosis, and response to treatment. A new strategy is now gradually complementing some gaps in this research field. Since the first Genome Wide Association Study (GWAS) was reported in Science in 2005 (Klein et al., 2005), there have been reports on cancer (Farashi et al., 2019), obesity (Loos and Yeo, 2014), type 2 diabetes (Langenberg and Lotta, 2018), and related phenotypes. A technologically highly feasible GWAS by identifying previously unrecognized genetic and epigenetic alterations might also improve understanding of the combination of genetic and acquired genetic and epigenetic changes critical for tumorigenesis. Therefore, it is crucial to understand the function of complex cancer-associated genetic alterations and epigenetic variants at the molecular level. Greater knowledge of how chromatin structure, DNA methylation, and gene expression affect cancer is progressively changing our view of carcinogenesis and cancer staging and classification. Genetic alterations and epigenetic modifications might become useful biomarkers for disease prognosis and treatment and will pave the way for truly personalized treatments and clinical applications for diagnosis, prognosis, and prevention.

Moreover, there is still much room to explore and review the understanding of genetic alterations and epigenetic modifications associated with cancer. Most clinical studies are cross-sectional or preliminary trials where the understanding of genetic alterations and epigenetic modifications has remained theoretical. Most of the current trials are limited to studying the effects of a particular epigenetic alteration on cancer. For example, Rivas et al. (2019) explored the potential mechanisms associated with aberrant epigenetic modifications in Hepatoblastoma. They found that the expression of genes associated with DNA methylation was in a generally disrupted state in Hepatoblastoma, demonstrating the role of epigenetic modifications in Hepatoblastoma progression. Rumbajan et al. (2013) analyzed the methylation status of 33 imprinted differentially methylated regions based on MALDI-TOF MS and pyrophosphate sequencing in 12 Hepatoblastomas and adjacent normal liver tissues and identified chromosomal abnormalities and frequent genetic and epigenetic alterations at the 11p15.5 and 20q13.3 sites. Large-scale cohort studies are needed to enrich these findings. Furthermore, genetic alterations and epigenetic modifications play a key role in the development and progression of Hepatoblastoma. To some extent, certain epigenetic modifications can be reversed under certain conditions, but there is a lack of more convincing evidence for their relationship. Moreover, most studies are often isolated and require greater support for further and deeper exploration.

In summary, with further research into Hepatoblastoma, the discovery of more genetic alterations and epigenetic modifications might provide new tools and targets for cancer prevention and treatment. Although the mechanisms of action are not yet elucidated and complex, genetic alterations and epigenetic modifications are crucial in cancer development.
